# First Schistosomal Cholecystitis Complicated by Cholangitis and Liver Abscess: Case Report and Review of Literature

**DOI:** 10.1155/2021/3470377

**Published:** 2021-07-12

**Authors:** Ali Toffaha, Samir Al Hyassat, Walid Elmoghazy, Hatem Khalaf, Ahmed Elaffandi

**Affiliations:** ^1^Department of Surgery, Hamad General Hospital, Doha, Qatar; ^2^Department of Laboratory Medicine and Pathology, Hamad General Hospital, Doha, Qatar; ^3^Department of Surgery, Sohag University, Cairo, Egypt; ^4^Department of Surgery, College of Medicine, Qatar University, Qatar; ^5^Department of Surgical Oncology, National Cancer Institute, Cairo, Egypt

## Abstract

Schistosomiasis is one of the most prevalent parasitic infections in the developing world. When it affects the gastrointestinal system specifically the liver, it causes periportal fibrosis followed by cirrhosis. Cholecystitis however is a rare presentation, and associated liver abscess has certainly never been reported to date. We report a case of acute cholecystitis complicated by cholangitis and liver abscess in a 46-year-old man. After complex course of treatment, he had laparoscopic cholecystectomy, and the histology report confirmed schistosomiasis. Gallbladder schistosomiasis is an uncommon disease that is associated with dense fibrotic changes that strongly mimics xanthogranulomatous cholecystitis. Liver abscess may occur during the disease evolution especially in patient originating from endemic backgrounds. We present the case and a comprehensive literature review.

## 1. Introduction

Parasitic infections remain a problem in the developing countries [1]. Schistosomiasis is responsible for more than 200 thousands deaths yearly [1]. Schistosomiasis may present acutely as febrile illness [2] or more commonly in chronic form due to eggs that are trapped in the tissues during the peri-vesical or peri-intestinal migration or after embolization in the liver, spleen, lungs, or cerebrospinal system [3]. Chronic lesions in these tissues are usually characterized by chronic inflammation and fibrosis which gives rise to the clinical manifestation of the disease (i.e., cirrhosis for hepatic involvement, chronic cystitis, and fibrosis for urinary involvement) [1].

In spite the fact of the high frequency of hepatic involvement particularly by Schistosoma *mansoni*, schistosomiasis of the gallbladder (GB) is remarkably uncommon [4]. About twelve cases have been retrieved from the literature. However, none of them was associated with liver abscess or cholangitis. A literature review in a comprehensive approach was carried out (Table 1). We report this case in line with the updated consensus-based surgical case report (SCARE) guidelines [5].

## 2. Case Presentation

A 46-year-old Egyptian man presented to the emergency department with one-day history of epigastric and right upper abdominal pain, associated with nausea and vomiting. He denied any other associated gastrointestinal or urologic symptoms. Apart from being type 1 diabetic, he declared no other significant past medical history.

**Figure 1:**
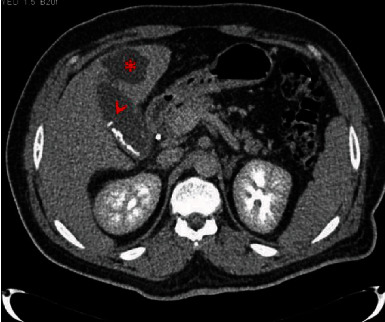
Abdominal CT scan showing gallbladder containing stones (arrow), with nearby segment IV b abscess (asterisk).

The patient presented in good shape, and there was no hemodynamic instability. He showed epigastric tenderness with no signs of peritonism with the rest of the abdomen unremarkable. White blood cell count (WBC) was 17.4 K/uL (4-10 K/uL). Liver function tests (LFTs) were abnormal: total bilirubin 47 umol/L (0-21 umol/L), direct bilirubin 35 umol/L (0-3 umol/L), ALT 156 U/L (0-40 U/L), and AST 182 U/L (0-41 U/L). Other results showed hemoglobin 14.4 g/dL (13-17 g/dL), platelets 190 K/uL (150-400 K/uL), lipase 106 U/L (13-60 U/L), CRP < 5 mg/L (0-5 mg/L), CA 19-9 303 U/ml (0-27 U/ml), and IgE 432 Ku/L (0-114 Ku/L). IgA and IgG4 were normal. Abdominal ultrasound (US) showed multiple GB stones with intrahepatic biliary dilatation and prominent common bile duct (CBD) measuring 7 mm. The rest of the examination showed no signs of acute cholecystitis and no bile duct stones. The patient was admitted as a case of obstructive jaundice for further work up. He later developed signs of sepsis (tachycardia and fever) for which blood cultures were taken. He was started on intravenous ceftriaxone and metronidazole. Endoscopic retrograde cholangiopancreatography (ERCP) showed purulent bile immediately following cannulation and failed to show any filling defects. Sphincterotomy and CBD stenting were done. ERCP procedure was not extraordinary in difficulty to suspect ampullary fibrosis or deformation. Magnetic Resonance Cholangiopancreatography (MRCP) later showed distended GB containing sludge and tiny stones, hyperenhancement of both GB and CBD walls, and mildly thickened GB wall in addition to pericholecystic edema and fat stranding, consistent with acute cholecystitis and cholangitis. There were no CBD stones nor thickening of the CBD wall. After stenting, the patient was kept on piperacillin/tazobactam. He improved clinically, and both his LFTs and inflammatory markers were trending down till the 4th day post-ERCP when he started to spike fever again. Septic work up was repeated, and endoscopic ultrasound (EUS) was done. This exam showed the stent in place. Both US and computed tomography (CT) showed a new lesion 5 × 4 × 5 cm in the segment IVb of the liver in continuation with the GB fundus (Figure 1). The lesion was compatible with a newly developed liver abscess. A percutaneous aspiration was carried out under US guidance, during which 100 ml of pus was aspirated and sent for microbiology/culture. During the aspiration, the GB was noticed to be deflating which pointed towards continuity between the liver abscess and the GB. Cultures grew Klebsiella oxytoca and Escherichia coli. The patient responded well after aspiration and antibiotic therapy and showed improved inflammatory markers. He was discharged the next day on oral antibiotics with close clinic follow-up to arrange for interval cholecystectomy after ERCP and stent removal.

**Figure 2:**
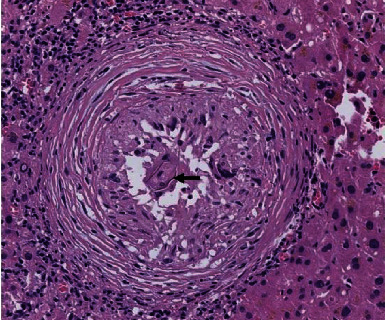
Microscopic image of gallbladder wall showing Schistosoma parasite within noncaseating granuloma (magnification HE ×20).

During follow-up visits, the patient was asymptomatic. ERCP was done 5 weeks after discharge and showed no filling defects in the CBD. The stent was removed. The patient travelled and was lost to follow-up for 5 months. When he came back, an MRCP showed complete resolution of the liver abscess. Multiple gall stones in GB were still demonstrated. He was booked for elective laparoscopic cholecystectomy. Intraoperative findings showed omental adhesions to the GB. The GB was also densely adherent to the liver. Despite the difficult dissection, the procedure was managed laparoscopically. Patient's postoperative course was unremarkable, and he was discharged next day after surgery. Histopathology of the gallbladder showed chronic cholecystitis, with granulomatous inflammation secondary to schistosomiasis (Figure 2).

## 3. Discussion

The first case of gallbladder schistosomiasis (GBS) was reported in 1975, and since then, speculations were made regarding possible pathogenesis [6]. Fourteen cases of GBS have been reported; however, none of them presented with associated complications. Few theories evolved on how schistosomiasis can cause cholecystitis. Some speculated that the fibrosis of the cystic duct, like what is seen in the ureters of patients with urinary schistosomiasis, causing a stenosis which can contribute to bile stasis and formation of stones in the gallbladder [7]. Others suggested that granulomatous inflammation in the gallbladder's wall makes it prone for stone formation [8].

The risk factor for contracting schistosomal infection is the contact of its larval form with the skin through contaminated water in endemic areas [4]. Most of the reported cases (Table 1) have been living at one stage in their life in an endemic area. Our reported case used to live in Egypt that is a well-known endemic area before moving abroad.

Clinical presentation is variable according to the involved organ. Infestation of urinary tract may lead to hematuria, fibrosis, and obstructive uropathy that may lead to parenchymal renal damage [1]. When it involves the liver, early inflammatory hepatic schistosomiasis happens in reaction to schistosomal eggs trapped in the presinusoidal periportal spaces of the liver. It then lead to typical features of sharp-edged enlargement of the liver nodular splenomegaly [1]. Intestinal involvement leads to diarrhea mostly due to mucosal granulomatous inflammation, pseudopolyposis, and microulcerations [1]. Reported symptoms of GBS are usually similar to other gallbladder diseases, including right upper quadrant pain that is sometimes associated with nausea and vomiting [7]. Abdominal examination shows right upper abdominal tenderness especially if the patient is having active cholecystitis (Table 1). The reported case first presented to the emergency with right upper quadrant abdominal pain. His disease progression was completely unique after GBS as he developed septic features due to cholangitis and associated liver abscess. This is, to our knowledge, the first reported case of cholecystitis with a liver abscess in a patient with schistosomiasis. This situation may however be as well secondary to a typical gallstone cholecystitis.

Cholangitis is mostly caused by ascending bacterial infection due to obstruction [9]. Causes of obstruction are variable (benign and malignant), the most common of which are biliary stones that usually slip from the gallbladder [9]. In our case, no stones were identified in the CBD by ERCP or MRCP; however, slipped stone to duodenum cannot be excluded as a probable cause. Another speculation is related to schistosomiasis pathogenesis leading to fibrosis and stricture of the CBD [10]. Despite early fibrotic changes of the CBD and/or ampulla being a possible cause in our case, however, we did not have any imaging evidence indicating gross CBD wall thickening or ampullary fibrosis.

Liver abscess has already been described as a complication of liver schistosomiasis [11]. However, it has not been yet reported as a part of the GBS disease progress (Table 1). Many speculations have been suggested to explain the link between schistosomiasis and liver abscesses. Bacteria tends to bind to laminin, fibronectin, and type IV collagen, which are plentiful in the active schistosomal granuloma. Moreover, the development and degradation of extracellular matrix of the granuloma may play a role on abscess formation [11]. Additionally, deposition of eggs was found to inhibit T cell response, so the usual immune response to bacteria can be affected [12]. Despite the mentioned speculations for liver abscess pathogenesis, cholecystitis and cholangitis itself can be responsible for this complication, as it is well reported cause for liver abscess whether concomitantly or remotely after resolution of cholecystitis [13]. Acute cholangitis and ERCP instrumentation are among other possible causes for liver abscess [14].

The imaging modality of choice for gallbladder disease is abdominal ultrasound, but it has no specific signs to indicate GBS [4]. In our case, the initial US showed gallstones which is similar to most of the reported cases seen in our review (Table 1). No specific blood tests are available for GBS. Only two cases showed positive serology after surgery (Table 1).

For GBS, the treatment remains surgical, usually by laparoscopic cholecystectomy [4]. At surgery, the gallbladder mimics cancer or xanthogranulomatous cholecystitis [15]. As per review, most cases showed irregularly thick and fibrosed gallbladder wall infiltrating into the liver at its bed alongside adhesions to the omentum and nearby bowel (Table 1). In the case presented, the fibrosis encountered in the liver bed could be secondary to schistosoma infestation, healing process secondary to the cholecystitis, or probably a combination of both. For timing of surgery, we preferred to proceed with an elective cholecystectomy after the patient passed the acute stage of disease and treatment for obstructive jaundice, cholangitis, and liver abscess. Elective cholecystectomy after cholangitis treatment is a feasible option as per Tokyo guidelines [16]. Other studies showed that drainage followed by delayed surgery is an acceptable treatment for cholecystitis concomitant with liver abscess [17, 18].

Moreover, four patients received postoperative complementary medical treatment which was praziquantel [8, 15, 19]. Similar to others, our patient received postoperative praziquantel.

The specimen pathology usually reveals a lymphocytic infiltrate; schistosomal eggs can be found in any layer of the gallbladder wall causing fibrocalcific reaction; most of the cases showed granulomatous inflammation surrounding the schistosomal eggs (Table 1).

## 4. Conclusions

GBS might be considered preoperatively in patients who lived in an endemic area and developed symptoms suggestive of gallbladder disease. This is the first case that report a liver abscess in a patient with cholecystitis with a gallbladder infested by Schistosoma. However, a majority of cholecystitis in patients with schistosomiasis involve the presence of gallstones. This condition carries the same possible complications and should be managed in the same way as usual cholecystitis. Surgeons must however expect a more difficult dissection during operation.

## Figures and Tables

**Table 1:** Summary of characteristics of current case and other reported cases of gallbladder schistosomiasis identified from the review of the literature.

Study^∗^	Sex	Age	pH	LEA	Presentation	D	PE	Labs	US	CT	Others	Surgery	Intra-op	Histo	F Up
Current study Qatar 2020	M	46 y	DM	Yes	RUQ pain	1 d	Epigastric tenderness	WBC: 17.4, Hb: 14.4, bili: 47, direct bili: 35, ALT: 156 U/L, AST: 182 U/L, lipase: 106 IU, CA 19-9: 303 U/ml, IgE: 432 *K* units/L, postop positive S serology	1st US: GS, Di IHBD, CBD: 7 mm 2nd US: new liver collection 4.3 × 3.2 cm	Newly developed liver abscess	MRCP: acute Chol, Cholang. ERCP: cholang, no filling defect, possibly narrow distal CBD	Lap Chole	Omental adhesions to the GB which was densely adherent to the liver	Chronic Chol, Gr Inf secondary to S	Prazi 40 mg/kg divided into 3 doses

Hedfi 2019 Tunisia [4]	F	51 y	DysL	No	Hepatic colic	2 m	N	N	Thin-walled GB, GS 10 mm	NR	NR	Lap Chole	Slightly thick-walled GB, fine cystic duct	Calcified S ova in the wall of GB stained positively for periodic acid-Schiff	CT urography: N

Majrashi 2018 Saudi [20]	M	50 y	DM	Yes	Elective surgery for biliary colic	9 y	RUQ tenderness	Positive S serology postop others: N	Wall thickness (4 mm), GS 8 mm	UR	NR	Lap Chole	Thick wall GB, with necrotic spots, firmly attached to the liver bed	Gr Inf around calcified S. haematobium eggs	Referred to ID team

Azoulay 2016 France [15]	M	53 y	NR	Yes	Elective after 2 episodes of Chol, recent 4 kg weight loss	5 m	N	N	Hyperechogenic thick GB wall, no GS	Thick GB wall 12 mm, contained calcifications and lesion protruding into GB and the liver, increased density of peri-vesicular fat, enlarged 2 hilar LN's (7 mm)	NR	Lap to open radical Chole (en bloc omental adhesions and LN resection)	Tense retraction of the right colon, duodenum, and omentum to the inferior aspect of the liver hampered Lap GB exploration	Acute and chronic Chol with dense fibrosis, S eggs in GB wall	Single dose of 2.4 mg of Prazi 15 d after surgery
Manes 2014 Greece [19]	M	77 y	NR	Yes	Elective 3 months after Chol	3 m	RUQ tenderness	N	Thick-walled GB (6.8 mm) GS 1.7 cm impacted at GB neck	NR	NR	Lap converted to open Chole	GB inflamed and thick with necrotic spots and wood-like consistency	Gr Inf around calcified S. mansoni eggs	Prazi 20 mg/kg every 4 h for 3 doses

Sharara 2001 Lebanon [8]	F	47 y	Smoker	No	RUQ discomfort	3 d	RUQ tenderness	AEC: 660/mm^3^ UA: Mic hem	Thick GB wall, 1 cm echogenic structure without acoustic shadow at GB fundus	Markedly thick GB wall, 2 hypodense liver lesions	NR	Lap Chole	Thick nondistended gallbladder firmly adherent to the liver surface and an enlarged cystic LN, no GS	Gr Inf around multiple S eggs, with the lateral spine, likely S mansoni	Prazi 20 mg/kg every 4 h for 3 doses

Bakhotma 1996 Saudi [21]	M	30 y		NR	RUQ pain, HU	NR	NR	UA: S. haematobium	GS	NR	NR	Lap Chole	Thickened wall	Chronic Chol with S. infection	Prazi, received before surgery

Al-Saleem 1989 Iraq [7]	M	27 y	NR	Yes	Biliary colic, hematemesis	2 m	Enlarged spleen down to the pelvis	NR	Huge spleen, thick GB wall, no GS	NR	OGD: varices lower two-thirds of the esophagus	L, Chole	Huge spleen, cirrhotic liver, GB grey, irregular in thickness, infiltrating into the liver bed. Thick cystic duct	Extensive S fibrosis	NR

Al-Saleem 1989 Iraq [7]	M	25 y	NR	Yes	Epigastric pain	2 m	NR	NR	Thick GB wall, large GS	NR	NR	Chole	Thick walled grey GB, the fibrosis so deep into the bed, thickened fibrotic, and calcified cystic duct	Extensive fibrocalcific GB S, due to S mansoni	NR

Al-Saleem 1989 Iraq [7]	M	62 y	Childhood HU	Yes	RUQ pain	NR	NR	NR	GS	NR	NR	Chole	Thick-walled grey GB, attached tightly to the liver and infiltrating it	Extensive fibrocalcific GB S, due to S haematobium	NR
Al-Saleem 1989 Iraq [7]	M	33 y	Childhood HU	Yes	Dull epigastric pain	3 m	NR	NR	Large GS	NR	NR	L, Chole	Thick-walled grey GB, with extensive fibrosis	Fibrocalcific GB S, due to S. haematobium	NR

Al-Saleem 1989 Iraq [7]	F	40 y	Obese	Yes	Dull RUQ pain	13 m	No tenderness	NR	Thick GB wall, large GS	NR	NR	Chole	Thick-walled grey GB, GS	Fibrocalcific GB S, due to S haematobium	NR

Al-Saleem 1989 Iraq [7]	M	55 y	NR	Yes	RUQ discomfort radiated to Rt shoulder, N&V	14 m	RUQ tenderness	NR	Thick GB wall, large GS	NR	NR	NR	Pancreatic tumour with multiple hepatic secondaries, thick-walled GB with stones	Biopsy showed extensive fibrosis, ova of S. haematobium	NR

Rappaport 1975 US [6]	M	51	NR	NR	RUQ pain, N&V, diarrhea	Few d	RUQ tenderness	N	NR	NR	IVP: N	Chole	Fibrotic liver, focally mildly thickened GB	Gr Inf, S. mansoni	NR

^∗^For space considerations, only the first author is cited. AEC: absolute eosinophil count; Bili: bilirubin umol/L; CBD: common bile duct; Chol: cholecystitis; Cholang: cholangitis; Chole: cholecystectomy; D: duration of symptoms; d: days; Di: dilated; DM: diabetes mellitus; DysL: dyslipidemia; F: female; F Up: follow-up treatment; GB: gallbladder; Gr: granulomatous; GS: gall stone/s; Hb: hemoglobin g/dl; HU: hematuria; ID: infectious diseases; IHBD: intrahepatic bile ducts; Inf: inflammation; Intra-op: intraoperative findings; IVP: intravenous pyelogram; L: laparotomy; Lap: laparoscopic; LEA: lived in an endemic area; LN's: lymph nodes; M: male; m: month/s; Mic: microscopic; N: normal; NR: not reported; N&V: nausea and vomiting; OGD: oesophagogastroduodenoscopy; PE: physical examination; post-op: postoperative; Prazi: praziquantel; Rt: right; RUQ: right upper quadrant; S: schistosoma/l; UA: urine analysis; UR: unremarkable; WBC: white blood cells K/uL; y: year/s.
